# Multifocal Amelanotic Melanoma of the Hard Palate: A Challenging Case

**DOI:** 10.3390/diagnostics10060424

**Published:** 2020-06-22

**Authors:** Luisa Limongelli, Eliano Cascardi, Saverio Capodiferro, Gianfranco Favia, Massimo Corsalini, Angela Tempesta, Eugenio Maiorano

**Affiliations:** 1 Department of Interdisciplinary Medicine, University of Bari “Aldo Moro”, Piazza G. Cesare 11, 70124 Bari, Italy; luisannalimongelli@gmail.com (L.L.); gianfranco.favia@uniba.it (G.F.); massimo.corsalini@uniba.it (M.C.); angelatempesta1989@gmail.com (A.T.); 2 Department of Emergency and Organ Transplantation, University of Bari “Aldo Moro”, Piazza G. Cesare 11, 70124 Bari, Italy; eliano20@hotmail.it (E.C.); eugenio.maiorano@uniba.it (E.M.)

**Keywords:** melanoma, head and neck, oral tumors, mucosal melanoma

## Abstract

Among all melanomas, the mucosal type is very rare and may occur in the sino-nasal mucosa, vagina, anus and the oral cavity. At variance with melanomas of the skin, no risk factors, such as familiarity, UV-exposure and skin phenotype, have been clearly identified for such neoplasms. Frequently, the diagnosis is delayed and achieved at advanced stages or when metastases have already occurred. The authors report on a case of mucosal melanoma of the oral cavity presenting as a mass of the hard palate in a 50-year old male, and the corresponding diagnostic-therapeutic pathway.

## 1. Introduction

Primary malignant melanoma (MM) supposedly originates from melanocytic precursors via the possible presence of pre-malignant lesions [1,2,3]. Less than 1% of all MM arise in the head and neck, the anterior maxilla and alveolar mucosa being the most frequently affected sites [2]. Males and females are equally affected, with no age predilection; also, ultraviolet (UV) exposure has been identified as a risk factor for cutaneous melanoma but it does not seem to play a role in MM, as applicable to alcohol drinking, cigarette smoking and irradiation [1,4,5]. The prognosis is usually poor, with a five-year survival rate of 30–35% and a median survival of 36 months [4,5]. Recently, many additional treatment options have been adopted for cutaneous melanoma, including targeted therapies and immunotherapies, which were proven effective to guarantee prolonged survival rates [3]; nevertheless, the application of such novel treatments to MM still is somehow limited by the low number of cases and the difficulties of performing adequate clinical trials.

Several cases of MM of the head and neck have been reported in the literature, but often no clear evidence showed whether such lesions could be considered primary or metastatic. This study reports on the salient clinical and histological features of one case of MM and highlights some peculiar immunohistochemical findings, which may be misleading when not accompanied by extensive sampling at distinct areas of the lesion.

## 2. Case Report

**Figure 1:**
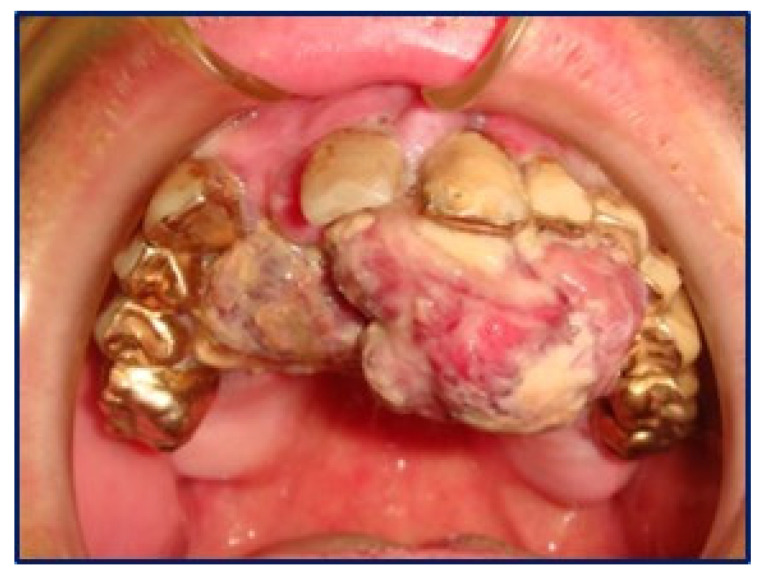
Rapidly growing lesion of the hard palate with periodontal involvement, with a red-yellowish appearance.

A 50-year old Caucasian male was referred to the Department of Odontostomatology of the University of Bari Aldo Moro (study n. 4599, prot. 1528/C.E; date of approval: 31 October 2014) with a three-month history of swelling and bleeding of the anterior maxillary area. His medical history was unremarkable, and the patient denied alcohol or smoking addiction. Oral examination revealed a firm and soft nodule, measuring approximately 4 × 6 cm, with pale appearance and focally ulcerated, extending bilaterally to the hard palate. In close proximity to the main nodule, black plaque and multiple pigmented lesions, involving the upper anterior gingiva were evident, which also extended towards and partly covered the non-removable dental prosthesis (Figure 1). No additional pigmented lesions could be detected elsewhere in the oral mucosa and subsequent dermatologic investigations excluded possible cutaneous primary lesions.

**Figure 2:**
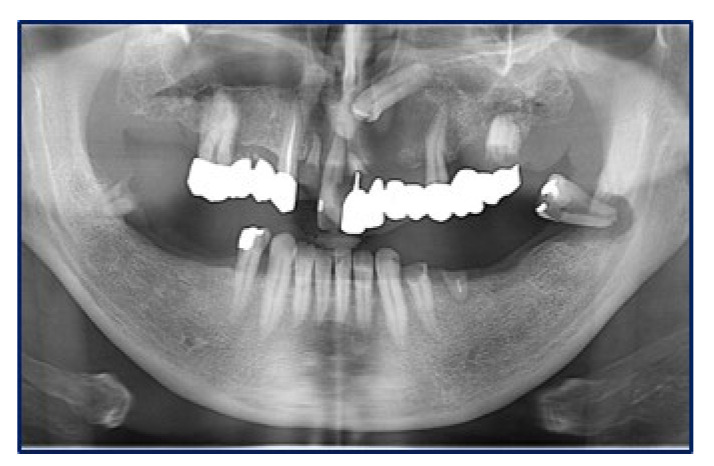
Panoramic radiogram showing an osteolytic lesion of the left anterior maxilla with undefined borders, involving the periodontal ligament of 2.1 and 2.4 as well as the retained 2.3 tooth.

**Figure 3:**
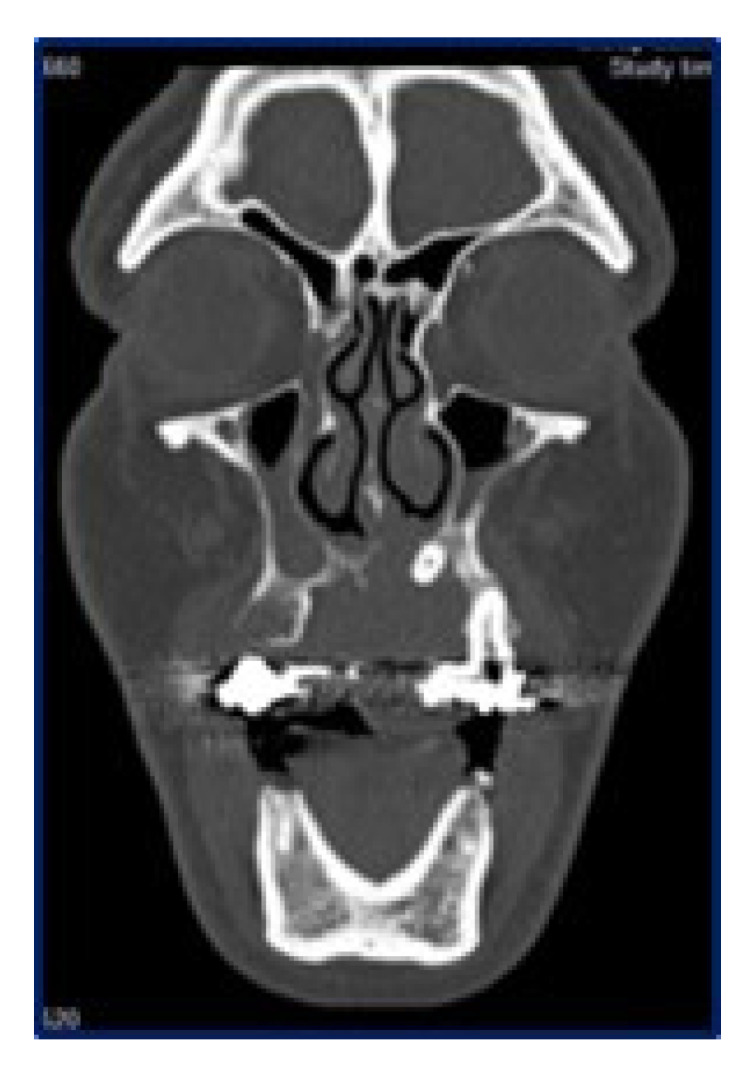
Sagittal computed tomography showing the extension of the lesion, with erosion of the hard palate and involvement of the maxillary sinus up to the orbital base.

Panoramic radiograms (Figure 2) and CT-scans (Figure 3) highlighted maxillary and palatal bone involvement, while US and MRI of the head and neck were negative for relevant loco-regional lymphadenopathy. A PET scan, brain CT and abdominal US showed no metastatic deposits. On such bases, the diagnostic hypothesis was of primary oral MM, clinically staged as T4aN0M0 [6].

**Figure 4:**
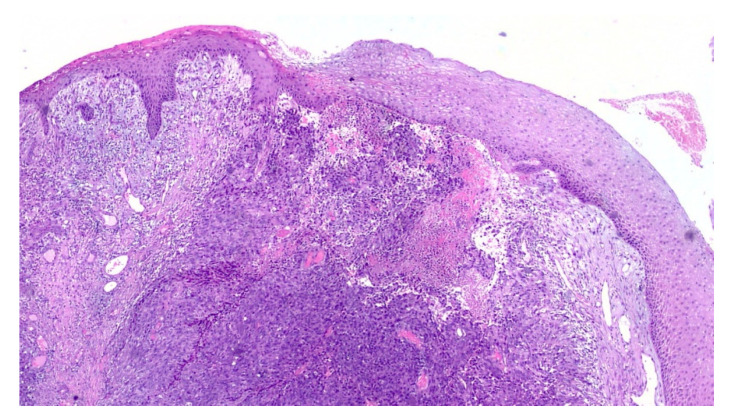
Low power view of a poorly demarcated lesion, located in the upper lamina propria (Hematoxylin & Eosin, ×2).

**Figure 5:**
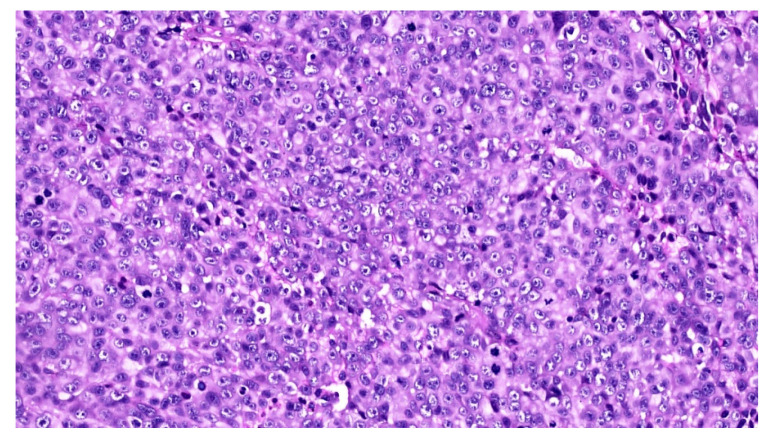
The tumor cells are epithelioid in shape, amelanotic and arranged in nests or nodules (Hematoxylin & Eosin, ×20).

An incisional biopsy of the mass was performed in an area without pigmentation, and the tissue fragment sent for histopathologic examination. Microscopically, the hematoxylin and eosin stained sections revealed a fragment of oral mucosa, lined by keratinized and hypotrophic stratified squamous epithelium (Figure 4), and a dense proliferation of spindle-shaped and epithelioid neoplastic cells in the lamina propria. The tumor cells displayed pleomorphic nuclei with prominent nucleoli and evident mitotic activity (Figure 5). Neoplastic junctional proliferation was detected in some areas.

**Figure 6:**
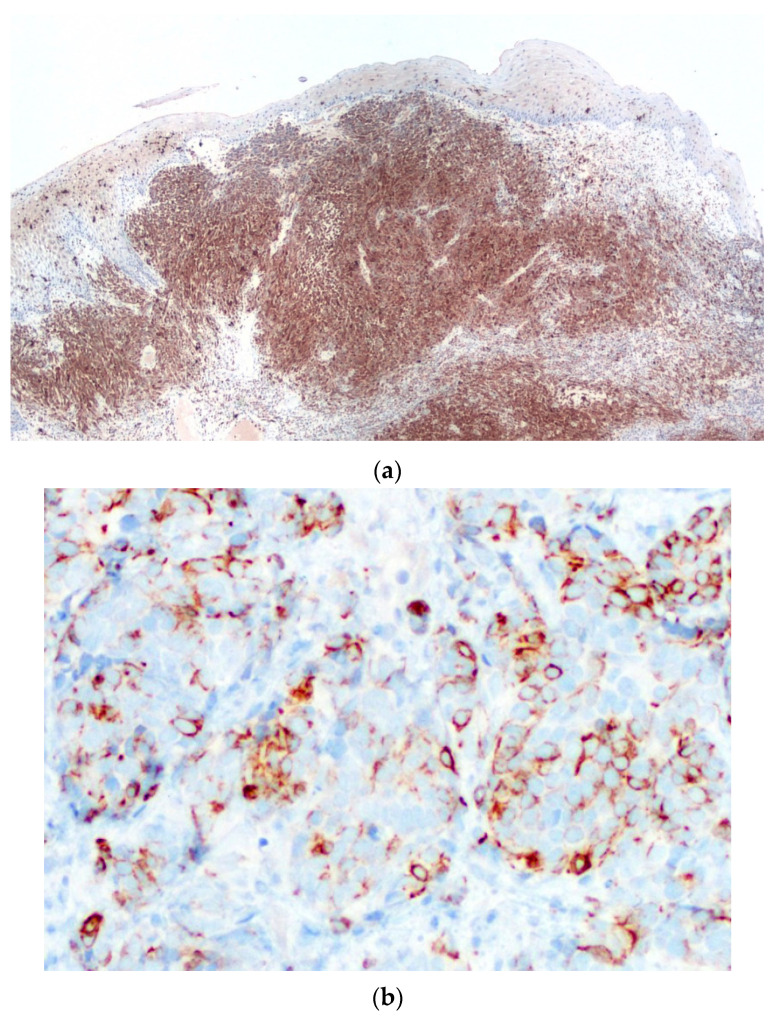

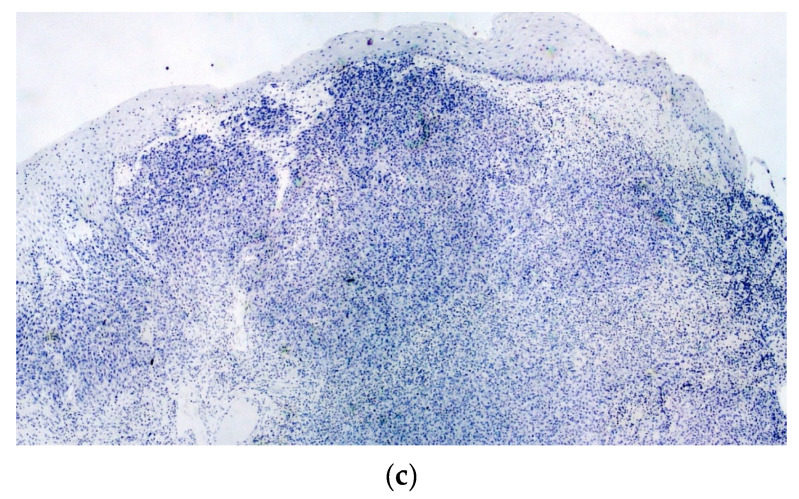
The neoplastic cells are strongly and diffusely immunoreactive for S-100 protein (**a**) (×2) and cytokeratins (with a dot-like pattern) (**b**) (×20), but negative for HMB-45 (**c**) (×2) as well as for Melan-A (not shown).

Immunohistochemical stains were performed to further characterize the neoplastic cell population, which highlighted pan-cytokeratins (with a dot-like pattern) and S-100 protein (Figure 6a) positivity, while HMB-45 (Figure 6b) and Melan-A were negative, thus leading to the diagnosis of malignant tumor, more consistent with MM. The tumor was subsequently treated by en-block resection with mapping-margins, the latter resulted tumor-free at histopathological evaluation. The postoperative period was uneventful.

**Figure 7:**
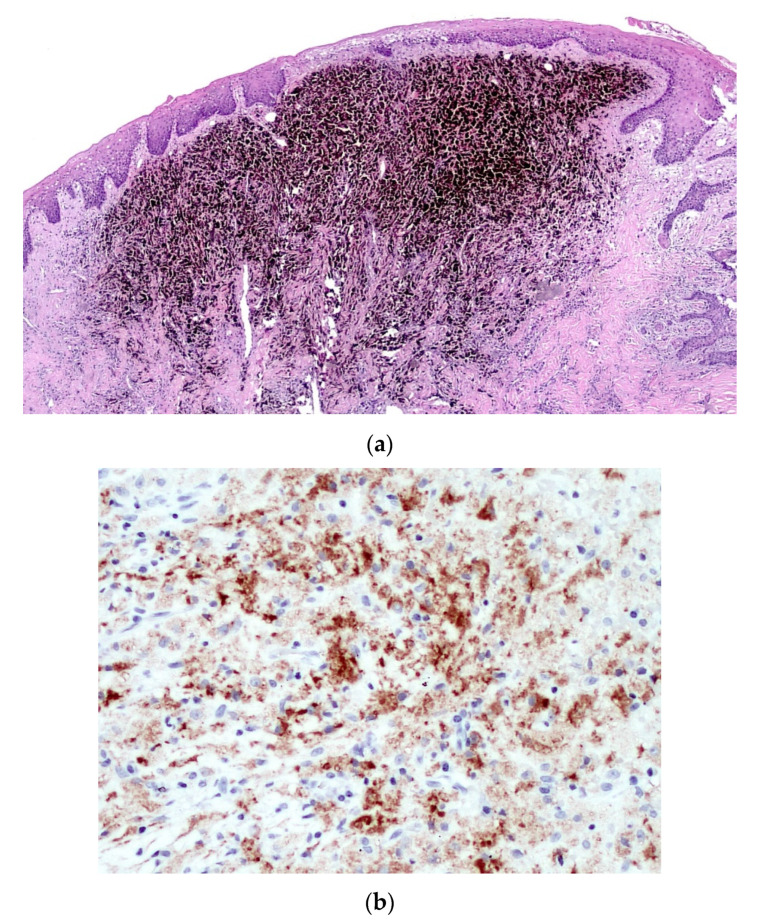
In this lesion, still located in the upper lamina propria, the tumor cells are filled with melanin pigment (**a**) (Hematoxylin & Eosin, ×2), and show consistent HMB-45 immunoreactivity (**b**) (×20).

An additional histopathological examination with much more extensive sampling was performed, showing an intra-mucosal hyper-melanotic lesion (Figure 7a) composed of spindle-shaped and epithelioid cells, displaying consistent S-100 protein immunoreactivity and less intense HMB-45 (Figure 7b) and Melan-A positivity. Such features lead to the final diagnosis of acral-type mucosal melanoma.

**Figure 8:**
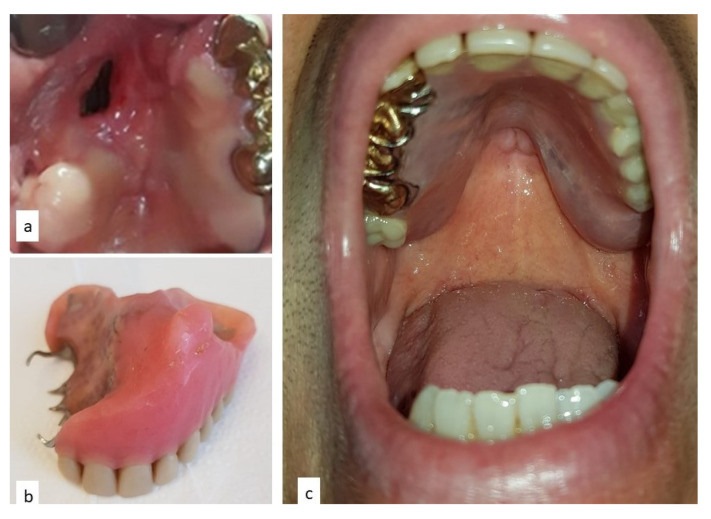
Post-operative defect of the palate with residual perforation: (**a**) receiving an obturator prosthesis (**b**) to improve chewing and protect airways (**c**).

The palatal mucosa healed with residual perforation (Figure 8a), and a palatal obturator was installed to improve chewing. The patient was then referred to the oncology unit and underwent adjunctive chemotherapy with two cycles of dimethyl triazeno-imidazole-carboxamide, nimustine hydrochloride and vincristine. Eleven months later, total body CT scans demonstrated metastatic lesions to the brain and the chest, which prompted two additional cycles of chemotherapy plus OK-432 and interferon 2; nevertheless, the patient died 30 months later with widespread disease.

## 3. Discussion

MM is very rare and represents 1–8% of all melanomas [7]. Such neoplasms may remain asymptomatic for a long time and, therefore, the final diagnosis may be achieved at later stages of the disease, with effacement of the prognosis, which usually is extremely poor [8]. The worst prognosis, in comparison with cutaneous melanoma, may depend on several factors, such as the diagnostic difficulty for mucosal pigmentations of this peculiar anatomical area and the consequent delayed diagnosis, the quicker and deeper infiltration of MM, thus making it hard to achieve disease-free excisional margins [9]. Furthermore, the differential diagnosis with non-malignant mucosal pigmentations may be extremely difficult, especially without high-resolution non-invasive imaging techniques (e.g., 400× dermoscopy/reflectance confocal microscopy/optical coherence tomography) [10].

Alcohol, cigarette smoking and formaldehyde exposure have been suggested as potential risk factors for oral melanomas [11,12], although none of these was reported by the patient. A primary oral melanoma may show variable clinical manifestations, including pigmented or amelanotic variants, and usually remains asymptomatic in the early stages. Surgical excision with supplementary radiotherapy is the most common treatment [13,14], but the recent immunotherapies seem promising to further ameliorate the prognosis.

Mucosal MMs usually are immunoreactive for S-100 protein and, less consistently, for HMB45 and Melan-A; also, previous reports highlight positivity for cytokeratin markers, although with variable distribution, diffuse staining being usually absent [15,16]. Mitotic activity frequently is prominent in MM, and Ki67 immunostain may be useful for the differential diagnosis with other melanocytic proliferations [6,17,18,19].

The latter include melanotic macule, amalgam tattoo, oral melanoacanthoma, oral nevus, poorly differentiated carcinoma and spindle cell/epithelioid sarcomas [20,21,22,23]. Melanotic macules consist in an increased number of pigmented cells in the basal layers of the epithelium, do not show a tumor-like shape or accumulations of tumor cells in the lamina propria [22]. Amalgam tattoo is characterized by the presence of brown-black pigment in the lamina propria, which may be freely accumulated among collagen bundles or within histiocytes [22]. Melanoacanthoma is an unfrequently detected lesion, characterized by a wide expansion of the epithelial compartment, due to the proliferation of epithelial cells, which accumulate intracellular melanin-like pigment. All these lesions may be easily ruled out, based on the absence of true melanocytic proliferation and the lack of S-100 protein, HMB45 and Melan-A immunoreactivity [22,23].

Oral nevi consist in accumulations of melanocytes either at the junctional area or within the lamina propria; at variance with MM, they display a symmetrical shape, and are composed by compact clusters of tumor cells, displaying minimal or no nuclear atypia and lacking mitotic activity [23]. Also, it is relevant to underline that the presence of in situ melanoma, junctional component or radial growth phase, though not always present, favor the diagnosis of primary MM and facilitate the exclusion of metastatic deposits. MM may show morphologic features similar to acral lentiginous melanoma, such as radial pattern of growth, pagetoid spread in the basal epithelium, fine and irregular pigment distribution and a nodular growth phase showing an admixture of epithelioid and spindle cell population, as was indeed the case in this instance. Nevertheless, though sharing many features with distinct subtypes of cutaneous melanoma (e.g., superficial spreading, nodular and acral), MMs are not usually subclassified as the peculiar clinico-morphologic features of such cutaneous subtypes do not seem to bear diagnostic or prognostic relevance for MM.

Poorly differentiated carcinomas may be more intriguing and difficult to rule out in view of their variable morphologic features and inconstant expression of epithelial markers, such as cytokeratins. Some of them may also show S-100-positivity, particularly those with myoepithelial differentiation; nevertheless, the use of additional epithelial markers, such as Epithelial Membrane Antigen and the lack of HMB45 and Melan-A immunoreactivity may be resolutive in this regard, the latter two antigen being usually absent in the vast majority of carcinomas and sarcomas, with few exceptions [14,15,21]. In this regard, we would like to emphasize that HMB45 and Melan-A, though more constantly detectable in skin melanomas, may show variable positivity in mucosal melanomas, as was indeed the case for the current case, at least in the sample which was initially submitted for intraoperative examination. Therefore, we would like to stress the need of a cautious attitude when facing an uncommon mucosal lesion in frozen sections, and ending up with a more generic provisional diagnosis of malignancy. The experience with the case reported here confirms that MM may show variable expression of melanoma-associated markers (i.e., HMB45 and Melan-A) in different areas of the lesion, more reproducible immunoreactivity for S-100 protein, and unexpected positivity for cytokeratins, thus suggesting the need for extensive sampling in different areas of the tumor to possibly highlight the unequivocal features of the melanoma, such as intra-cellular pigment deposits, evident nuclear atypicality with prominent nucleoli, and intense mitotic activity, which may be unevenly distributed throughout the neoplasm [24].

## 4. Conclusions

Primary MMs of the oral cavity are very rare and, usually, their occurrence is asymptomatic in the early stages, thus frequently leading to a delayed diagnosis. Also, as their morphological features are often misleading or non-specific, the diagnosis must rely upon accurate histopathological investigation and extensive immunohistochemical evaluation too. It is worth emphasizing that frequently melanocyte-specific antigens (Melan-A and HMB-45) are unevenly distributed in MMs, and unexpected cytokeratin positivity may occur, which may result in an inaccurate diagnosis.
